# Candidate High-Resolution Mass Spectrometry-Based Reference Method for the Quantification of Procalcitonin in Human Serum Using a Characterized Recombinant Protein as a Primary Calibrator

**DOI:** 10.1021/acs.analchem.1c03061

**Published:** 2022-03-02

**Authors:** Huu-Hien Huynh, Vincent Delatour, Maxence Derbez-Morin, Qinde Liu, Amandine Boeuf, Joëlle Vinh

**Affiliations:** † Department of Biomedical and Organic Chemistry, Laboratoire National de Métrologie et d’Essais (LNE), 75724 Paris, France; ‡ Biological Mass Spectrometry and Proteomics, SMBP, PDC UMR 8249 CNRS, ESPCI Paris, Université PSL, 75005 Paris, France; § CEA, INRAE, Département Médicaments et Technologies pour la Santé (DMTS), SPI, Université Paris-Saclay, 91191 Gif-sur-Yvette, France; ∥ Chemical Metrology Division, Applied Sciences Group, Health Sciences Authority, 117528 Singapore

## Abstract

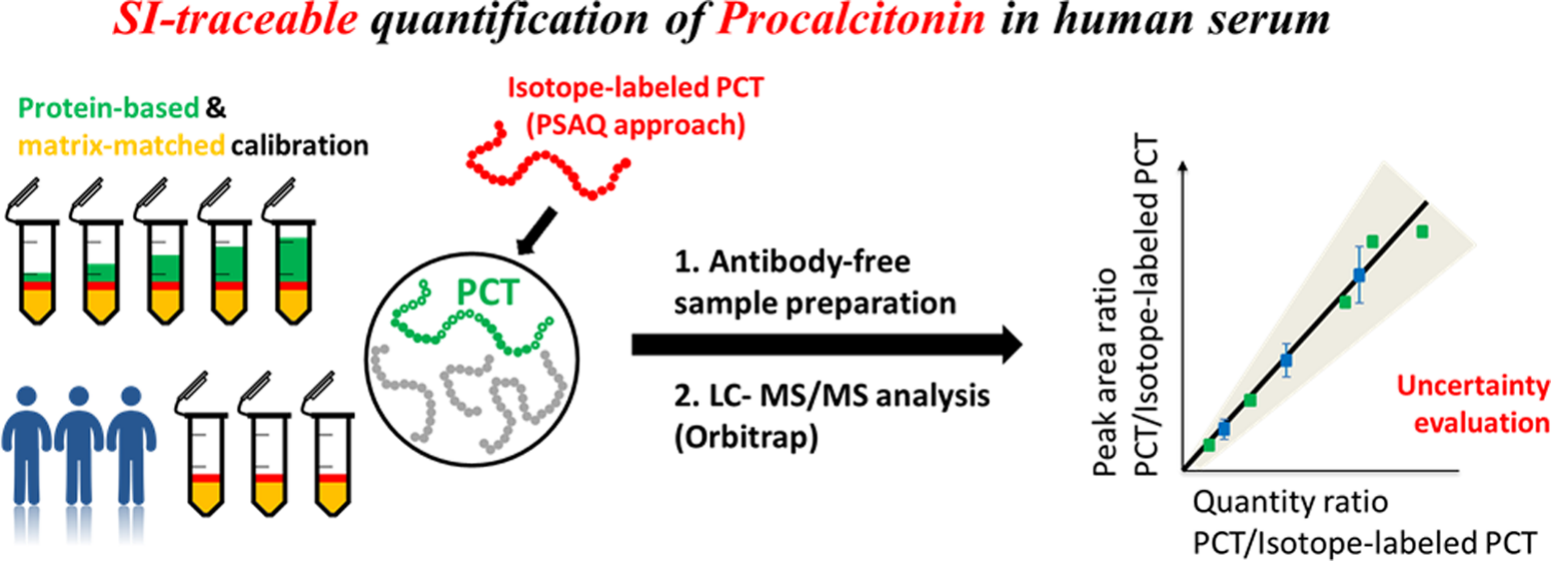

Procalcitonin (PCT) is a widely used biomarker for rapid sepsis diagnosis and antibiotic stewardship. Variability of results in commercial assays has highlighted the need for standardization of PCT measurements. An antibody-free candidate reference measurement procedure (RMP) based on the isotope dilution mass spectrometry and protein calibration approach was developed and validated to quantify PCT in human serum. The method allows quantification of PCT from 0.25 to 13.74 μg/L (*R* > 0.998) with extension up to 132 μg/L after dilution of samples with PCT concentration above 13.74 μg/L. Intraday bias was between −3.3 and +5.7%, and interday bias was between −3.0 and −0.7%. Intraday precision was below 5.1%, and interday precision was below 4.0%. The candidate RMP was successfully applied to the absolute quantification of PCT in five frozen human serum pools. A recombinant PCT used as a primary calibrator was characterized by high-resolution mass spectrometry and amino acid analysis to establish traceability of the results to the SI units. This candidate RMP is fit to assign target values to secondary certified reference materials (CRMs) for further use in external quality assessment schemes to monitor the accuracy and comparability of the commercially available immunoassay results and to confirm the need for improving the harmonization of PCT assays. The candidate RMP will also be used to evaluate whether the correlation between the candidate RMP and immunoassays is sufficiently high. Overall, this candidate RMP will support reliable sepsis diagnosis and guide treatment decisions, patient monitoring, and outcomes.

Procalcitonin (PCT) is a recognized sepsis biomarker allowing patient stratification and antibiotic therapy management.^1−3^ Different clinical decision cut-offs were established (e.g., 0.5 μg/L for sepsis diagnosis and 0.25 μg/L for antibiotic initiation or discontinuation for a patient with moderate or mild illness outside ICU^4^). PCT measurement has been integrated into clinical guidelines and antimicrobial stewardship programs.^4−6^ Thus, reliable and accurate measurements of this biomarker are critical for sepsis diagnosis, guiding treatment decisions, and patient monitoring. Facing a growing demand for PCT testing, the number of commercialized assays based on different technical principles has increased considerably in recent years.^7^ Different studies underlined discrepancies of results provided by various commercially available PCT assays.^8−11^ These discrepancies may impact clinical decisions at cut-offs, leading to disease misclassification and inappropriate antibiotic treatment decision. However, the source of such variability remains unclear.^12^ A proposed route to improve comparability and accuracy of the results is developing reference calibration materials, which have been value-assigned with a higher-order reference measurement procedure (RMP).^13−15^ Such a higher-order reference measurement system is still missing for PCT. Some assays were harmonized through traceability to the Brahms PCT LIA assay, but this protocol was not adopted for all assays. Moreover, the traceability of the results to SI units has not yet been established. Having such a higher-order measurement system will pave the road toward the standardization/harmonization of PCT assays, which has been considered a high priority by the International Consortium for Harmonization of Clinical Laboratory Results.^16^ As a first step, an RMP would help confirm the need to improve PCT assay harmonization and evaluate if the correlation with the commercially available PCT immunoassays is suitable for standardization. In addition, an RMP will support the establishment of traceability of results to a higher-order reference, as required by ISO 17511:2020 and the European regulation 2017/746 for in vitro diagnostic devices.^17,18^

Thanks to their high selectivity and reproducibility, isotope dilution and mass spectrometry have been successfully implemented to develop RMPs for SI-traceable quantification of clinically relevant proteins.^19−21^ Three studies based on isotope dilution associated with liquid chromatography tandem mass spectrometry (ID-LC-MS/MS) were previously reported for PCT quantification in serum.^22−24^ Each relied on stable isotope labeled (SIL) peptides spiked in the sample after protein digestion. However, a SIL protein, spiked at the earliest stage of the sample preparation to overcome material loss or variability occurring during sample processing and digestion, is considered an ideal internal standard with the same behavior as the analyte of interest.^25−28^

Here, we described the development and validation of a candidate reference ID-LC-MS/MS method for the SI-traceable quantification of PCT in serum at clinically relevant concentrations using, for the first time, a recombinant protein as a primary calibrator and a SIL-recombinant protein as an internal standard (Figure 1). In addition, analytical performance in terms of trueness and precision was assessed, and the uncertainty of measurement results was evaluated. Finally, the present method was used to perform SI-traceable quantification of PCT in five pools of frozen human serum as a proof of concept for developing secondary certified reference materials (CRM).

**Figure 1:**
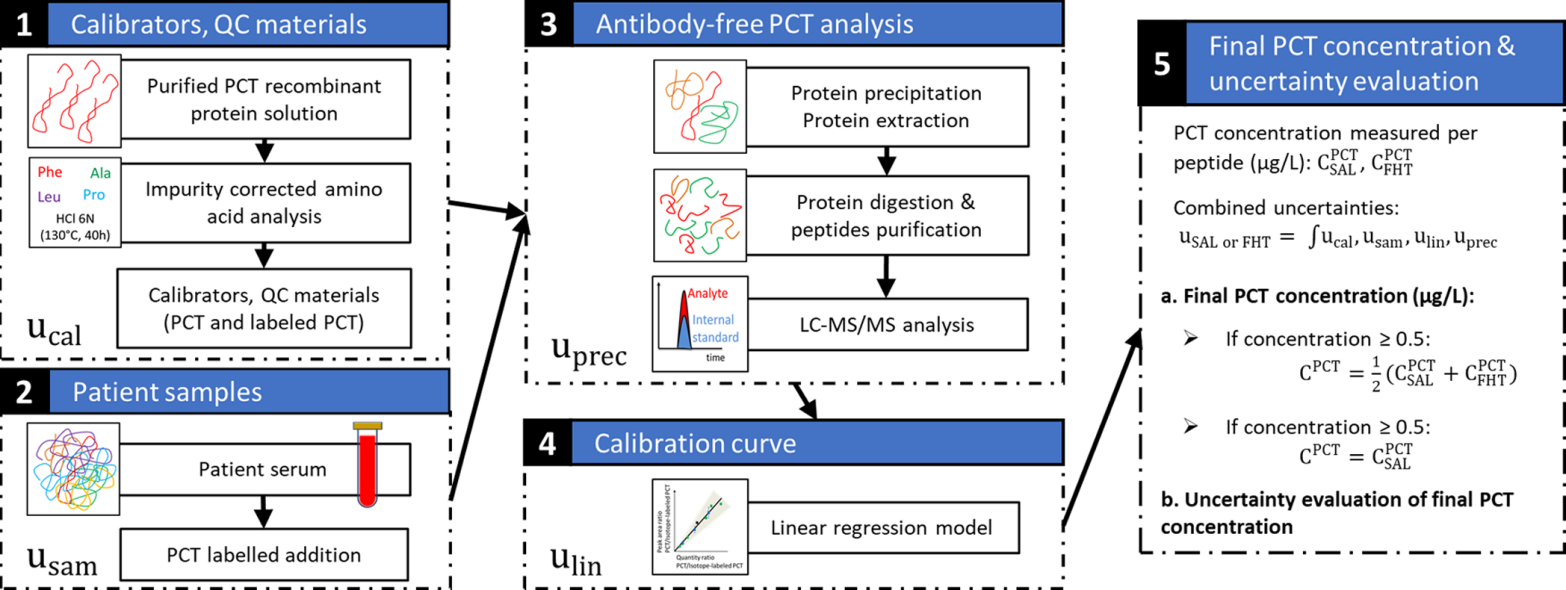
Schematic analytical workflow for SI-traceable quantification of PCT in human serum and its uncertainty using protein-based matrix-matched calibration and labeled PCT recombinant protein as internal standard. Step 1: Preparation of calibrators and quality control (QC) materials in blank serum using the SI-traceable PCT primary calibrator after performing impurity-corrected amino acid analysis (AAA). Step 2: Preparation of patient samples by spiking labeled PCT. Step 3: Antibody-free sample preparation for calibrators, QC materials, and patient samples followed by LC-MS/MS analysis of final processed samples. Step 4: Establishment of a calibration curve using a linear regression model to determine PCT concentration measured per peptide SAL or FHT. Step 5: Determination of PCT concentration based on two selected peptides, and its associated uncertainty was estimated by combining all sources of uncertainty from steps 1 to 4 (u_cal_, u_sam_, u_prec_, u_lin_).

## Experimental Section

### Chemicals and Reagents

Amino acid CRMs from NMIJ, chemicals, and reagents were described in a previous study^24^ and are detailed in document 1 of the Supporting Information.

The recombinant protein methionine-procalcitonin 3–116 (Met-PCT [3–116]) and the isotopically labeled protein methionine-procalcitonin 3–116 (SIL protein Met-PCT [3–116] labeled on arginine (R[^13^C_6_,^15^N_4_]) and lysine (K[^13^C_4_,^15^N]) residues) at a concentration of ∼1 g/L (Tris/NaCl buffer solution) were purchased from Promise Advanced Proteomics (Grenoble, France). The supplier purified Met-PCT [3–116] using three orthogonal techniques: ion exchange, reverse-phase, and size-exclusion chromatography.

### Instrumentation

Amino acid analyses, intact mass LC-MS measurements of the primary calibrator Met-PCT [3–116], and LC-MS/MS analyses of the digested serum samples were performed on a Thermo Scientific Dionex Ultimate 3000 ultraperformance liquid chromatography system coupled to a Thermo Scientific Q Exactive Focus hybrid Quadrupole-Orbitrap mass spectrometer (Thermo Scientific, Waltham, MA).

The top-down analysis of Met-PCT [3–116] was conducted on a Thermo Scientific Dionex RSLC Ultimate 3000 nano-LC system coupled to a Thermo Scientific Orbitrap Eclipse Tribrid mass spectrometer (Thermo Scientific).

### Sample Collection

University Hospital Montpellier (Montpellier, France) provided five pools of deidentified patient serum samples with different PCT concentrations. Each pool was produced by pooling 12 single frozen leftovers (collected in dry tubes) obtained from sepsis or septic shock patients. PCT concentration of these serum pools was determined at the clinical chemistry laboratory of Montpellier Hospital using the Brahms PCT sensitive Kryptor immunoassay (Compact Plus). The serum pools were then immediately stored at −80 °C ± 10 °C until analysis.

### Characterization and Quantification of Primary Calibrator Stock Solution

#### Confirmation of Met-PCT [3–116] and Impurity Analysis

Met-PCT [3–116] protein and its impurities were characterized using two complementary approaches: high-resolution MS analyses of intact protein for impurity identification and top-down MS analyses using multiple fragmentation modes for protein characterization.

##### LC-MS Conditions for Intact Protein Analysis

A Met-PCT [3–116] solution (∼0.1 g/L in H_2_O/ACN 95:5, v/v) was analyzed for potential impurity identification in LC-MS, operated in electrospray positive mode (Q Exactive Focus). LC was performed on a C4 analytical column (150 mm × 1 mm, 5 μm, BioBasic-4, Thermo Scientific). The mobile phase consisted of 0.1% FA (v/v) in water (solvent A) and 0.1% (v/v) FA in acetonitrile (solvent B). The separation was achieved using a linear gradient from 25 to 60% of B over 37 min at a 40 μL/min flow rate. The experimental MS parameters are summarized in Table S1 of the Supporting Information.

##### LC-MS/MS Conditions for Top-Down Protein Analysis

The Met-PCT [3–116] solution at 0.1 g/L was also analyzed on a nanoelectrospray tribrid Eclipse instrument. LC separation was performed on a C4 analytical column (75 μm × 150 mm, 5 μm, Acclaim PepMap 300, Thermo Scientific). The mobile phase consisted of H_2_O/ACN 98:2 (v/v), 0.1% FA (solvent A) and H_2_O/ACN 10:90 (v/v), 0.1% FA (solvent B). The separation was achieved using a linear gradient from 25 to 60% B in 37 min at a 300 nL/min flow rate. The sample was analyzed in data-dependent acquisition mode, using four different fragmentation modes (HCD, EThDC, CID, and UVPD). The experimental MS parameters are summarized in Table S2 of the Supporting Information.

#### Amino Acid Analysis (AAA)

The SI-traceable quantification of Met-PCT [3–116] standard was performed by amino acid analysis (AAA) as described previously.^24^ Briefly, the Met-PCT [3–116] content was determined by quantifying phenylalanine, proline, valine, and leucine by ID-LC-MS using a five-point calibration curve after gas-phase hydrolysis in acidic conditions. Conditions of the gas-phase hydrolysis were optimized by carrying out the gas-phase hydrolysis (ELDEX Workstation) at different conditions: 110, 130, or 150 °C for 40 h and 130 °C for 24 and 72 h. In each condition, four processed replicates were performed. The amino acid mix was analyzed on the Q Exactive Focus instrument in the selected ion monitoring mode. Isocratic separation was performed using a C18 column (150 mm × 2.1 mm, 1.7 μm, BEH C18, Waters) in H_2_O/ACN/FA 98:2:0.1 (v/v/v). The final protein concentration was estimated as the average of the four amino acid titrations determined from optimal hydrolysis conditions with 29 processed replicates over six independent experiments.

### Preparation of Calibration and QC Materials

Calibration and quality control (QC) materials were blank serum samples spiked with the recombinant Met-PCT [3–116]. Detailed preparation is available in document 2 of the Supporting Information. Briefly, a set of six calibration samples (concentration of Met-PCT [3–116] ranging from ∼0.25 to 13.74 μg/L) and three QC samples (concentration of Met-PCT [3–116] 1.0, 4.0, and ∼9.0 μg/L) was prepared by spiking Met-PCT [3–116] at different concentrations and SIL Met-PCT [3–116] at ∼1.7 μg/L in blank serum gravimetrically. The mass ratio between unlabeled and SIL protein ranged from ∼0.15 to 7.5 for calibration materials and 0.51–5.08 for QC materials. The calibration and QC materials were aliquoted (500 μL) and stored at −80 °C ± 10 °C .

QC materials used to determine the lower limit of quantification (LLOQ) were prepared by spiking Met-PCT [3–116] at two different concentrations (∼0.25 and ∼0.50 μg/L) and SIL Met-PCT [3–116] at ∼1.7 μg/L in blank serum. The mass ratios between unlabeled and SIL protein were 0.15 and 0.30.

QC materials used to determine the higher limit of quantification (HLOQ) were prepared by spiking Met-PCT [3–116] at ∼132 μg/L in blank serum. The sample was then diluted in blank serum to a concentration of ∼6.5 μg/L followed by the addition of SIL Met-PCT [3–116] at ∼1.7 μg/L. The mass ratio between unlabeled and SIL protein was 3.8.

### Sample Preparation Procedure for PCT Quantification in Human Serum

Patient samples were prepared gravimetrically by mixing about 480 μL of the sample with 20 μL of SIL Met-PCT [3–116] at a concentration of ∼40 μg/L to reach a final concentration of ∼1.5 μg/L. The calibration, QC materials, and patient samples were processed as described previously.^24^ Briefly, 500 μL of serum was subjected to protein denaturation using SDC detergent and precipitated using acetonitrile. Next, the supernatant was diluted and purified on a C18 solid-phase extraction (SPE) cartridge (HLB C18, Waters). Extracted proteins were reduced (DTT), alkylated (IAA), and digested with 4.6 μg trypsin gold. Finally, the tryptic digest was purified on an HLB C18 SPE cartridge. The elution buffer was evaporated to dryness in a centrifugal vacuum concentrator, reconstituted with 100 μL of 0.1% formic acid, 2% MeOH in water (v/v/v) (noted final extract), and stored at −20 °C ± 5 °C until LC-MS/MS analysis.

### LC-MS/MS Conditions

The proteolytic digests were analyzed in parallel reaction monitoring (PRM) mode^29^ on the Q Exactive Focus instrument. Briefly, tryptic peptides were separated on a C18 analytical column (1 mm × 150 mm, 3 μm, Acclaim PepMap 100, Thermo Scientific) using 0.05% AA in water (v/v) as solvent A and 0.05% AA in methanol (v/v) as solvent B at a flow rate of 80 μL/min. Peptides were eluted with the following gradient of mobile phase B: 2% for 2 min, linear from 2 to 22% in 8 min, linear from 22 to 38% in 1 min, linear from 38 to 42% in 14 min, and from 42 to 98% in 1 min.

SALESSPADPATLSEDEAR (noted SAL) and FHTFPQTAIGVGAPGK (noted FHT) proteotypic peptides have been previously selected for PCT quantification.^24^ Two transitions per peptide were selected, one used as peptide quantifier and another as peptide qualifier (see Table S3, Supporting Information). Raw data were processed with Xcalibur software v4.1 (Thermo Scientific). Signal extraction in the LC profile was performed within a mass tolerance of 10 ppm for PRM data.

### Method Validation

After defining the calibration curve, the analytical performance for PCT quantification in human serum using a protein-based calibration approach with SIL protein as internal standard was validated based on matrix-matched material according to FDA and EMA guidelines^30,31^ regarding linearity, trueness, precision, dilution, autosampler stability of extracted peptides, and carryover. The trueness and precision were performed using matrix-matched QC materials in three processed replicates over three independent experiments using freshly prepared calibrators for each experiment. Protocol and criteria for method validation are described in document 3 of the Supporting Information.

### Uncertainty Evaluation of PCT Quantification in Human Serum

Uncertainty was evaluated according to the ISO Guide 98-3GUM using the bottom-up approach.^32^ The combined uncertainty of the experimental values for QC, LLOQ, HLOQ levels, and patient pools for individual concentration obtained per peptide (u_SAL_ and u_FHT_) was calculated by propagating the uncertainty associated with all relevant sources of measurement uncertainty, including primary calibrator uncertainty and gravimetric preparation of calibrators (u_cal_), gravimetric preparation of samples (u_sam_), regression model (u_lin_), and intermediate precision (u_prec_).

The uncertainty (u_mean_) of mean concentration was calculated by combining the uncertainties of two individual concentrations per peptide.
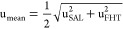
The final uncertainty (u_final_) of mean concentration was calculated by taking into account the uncertainty between peptides (u_interpeptide_) obtained from analysis of variance (ANOVA).
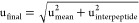
Finally, the expanded uncertainty (U) was expressed by multiplying the final uncertainty with a coverage factor *k* = 2, corresponding to a confidence level of ∼95%. The relative expanded uncertainty (%) was expressed by the ratio between the expanded uncertainty and the measurement result.

## Results and Discussion

Developing a candidate reference measurement to quantify PCT in human serum requires each analytical process step to be metrologically traceable to SI units. Figure 1 illustrates the workflow for SI-traceable quantification of PCT in human serum of the developed method.

### Characterization and Quantification of Primary Calibrator Stock Solution

#### Confirmation of Met-PCT [3–116]

**Figure 2:**
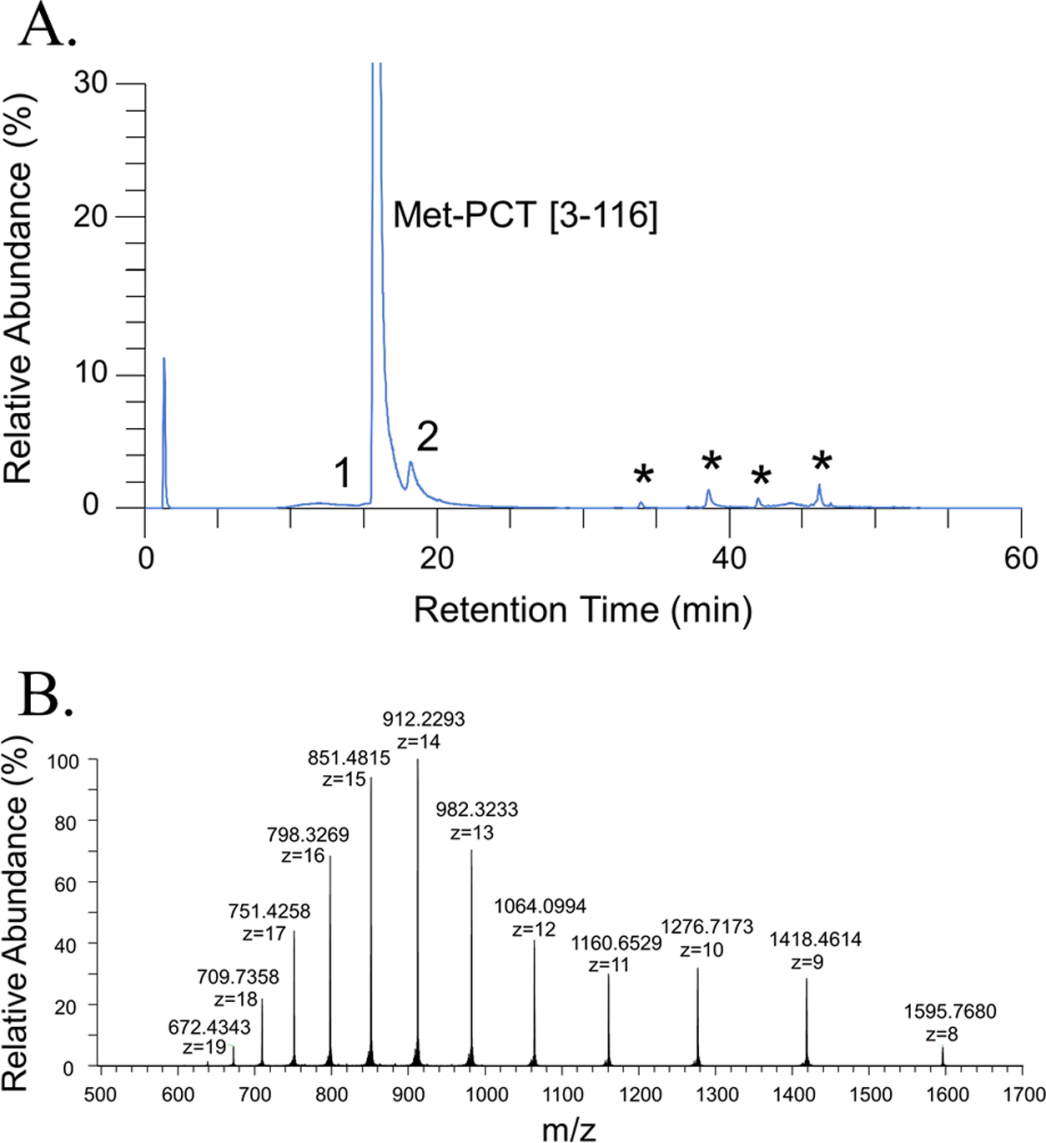
Characterization of the Met-PCT [3–116] primary calibrator. (A) Extracted ion chromatogram obtained by injecting 5 μg of the protein standard. The base peak represents Met-PCT [3–116]. 1—Oxidized form; 2—acetylated form; and *—monocharged compounds. (B) Multicharged mass spectrum corresponding to the base peak Met-PCT [3–116] at 15.8 min.

A total ion chromatogram obtained after LC-MS analysis of the primary calibrator is presented in Figure 2A. The MS spectrum corresponding to the major chromatographic peak at 15.8 min is presented in Figure 2B. A monoisotopic mass of 12 749.12 Da was identified, which agreed well with the theoretical value of Met-PCT: 12749.11 Da (Δ_mass_ −0.39 ppm). The identity of Met-PCT [3–116] was also confirmed by top-down MS/MS analysis. By combining four fragmentation modes on the charge state 14 of the major compound in buffer stock solution (*m*/*z* = 912.2293), 60% of Met-PCT [3–116] sequence coverage was obtained and the identity of the major compound in the buffer stock solution of the primary calibrator was confirmed (see Figure S1, Supporting Information).

#### Impurity Analysis

The analytical challenge of developing a protein-based primary calibrator is identifying and quantifying all impurities impacting either AAA or LC-MS/MS quantification of PCT in a matrix, which can be burdensome. To limit this issue as much as possible, the primary protein calibrator should be highly purified. Upon request to the Promise manufacturer, the recombinant PCT protein was subjected to three orthogonal chromatographic strategies: ion exchange, reverse-phase, and size-exclusion chromatography. However, as some impurities are similar (e.g., proteoforms), a 100% pure recombinant protein is almost unattainable, even with a high cost in terms of yield. Figure 2A shows the presence of additional peaks around the peak of Met-PCT. The most intense impurities identified by accurate mass measurement were oxidized PCT, acetylated PCT, and four truncated forms of PCT (see Table S6, Supporting Information). The associated peak area obtained from extracted chromatogram after deconvolution of different species was compared. The relative areas of oxidized PCT and acetylated PCT (in the stock solution) peaks correspond to 5.07 and 2.14% of the Met-PCT peak area.

The relative peak areas of truncated forms of PCT were less than 0.6%. The top-down analysis confirmed that acetylation occurs on one of the three N-terminal residues of PCT (Met–Phe–Arg). Thus, this impurity affects neither the AAA results nor the LC-MS/MS quantification of the targeted peptides SAL or FHT. Regarding the oxidized form of Met-PCT, it was not yet possible to unambiguously identify the oxidation site based on top-down analysis. However, no oxidized form of peptide SAL or FHT (±5 min from retention time of peptide SAL or FHT) was detected based on LC-MS analysis of samples after trypsin digestion of Met-PCT in the buffer. Moreover, the most frequent residues subject to oxidation are methionine and cysteine: they are not among the residues targeted by AAA (phenylalanine, proline, valine, and leucine), and they are not found in the two targeted peptide sequences. The truncated forms observed with a delta mass of about −1300 Da had a retention time close to the recombinant protein one. The purification steps performed by the supplier of the recombinant protein, including size-exclusion chromatography, suggest that these low abundant truncated forms were artifacts generated during the LC-MS analysis and were not present in the original sample. The two modified forms with delta mass of +29 and −17 Da coeluted with the recombinant protein. The absence of chromatographic separation of these modified forms from the recombinant protein when using different elution gradients also suggests that these low abundant forms are artifacts generated during the LC-MS analysis. Therefore, the raw amino acid analysis results were not corrected, highlighting the benefits of working with highly purified materials.

#### Quantification of Primary Calibrator Stock Solution by AAA

AAA determined the concentration of the primary calibrator to establish the traceability of the results to the SI units. After optimizing the conditions of gas-phase hydrolysis, the highest concentration measured by AAA, with the lowest variation between the four amino acids (leucine, phenylalanine, proline, valine), was obtained at 130 °C for 40 h (see Table S4, Supporting Information). These experimental conditions allowed hydrolyzing the valine amide liaison, challenging to cleave without degrading the amino acids produced. These optimized conditions were then applied to the quantification of the four amino acids in the primary stock solution of Met-PCT [3–116] (*N* = 29). The mass fraction of Met-PCT [3–116] (average from four amino acid results) in the stock solution was 807 ± 72 μg/g (*k* = 2) (see Table S5, Supporting Information).

### Method Validation

**Figure 3:**
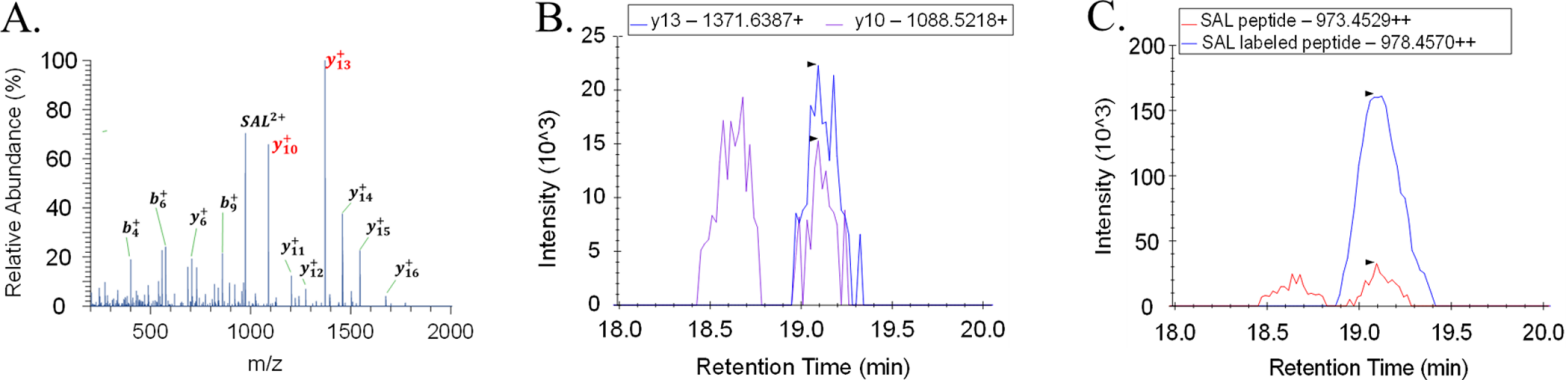
Identification of the SAL peptide for PCT quantification in human serum. (A) MS/MS PRM spectrum of targeted precursor ion SAL^2+^ (selected product ions y_13_^+^ and y_10_^+^ for quantification and confirmation in red) in processed human serum spiked with a PCT at 5 μg/L; (B) extracted ion chromatograms obtained when measuring blank serum spiked with a PCT at LLOQ level showing coelution of two selected product ions; (C) extracted ion chromatograms obtained when measuring blank serum spiked with a PCT at the LLOQ level and labeled PCT at 1.5 μg/L showing coelution of the SAL peptide and its internal standard. Precursor ions were isolated within an isolation window of 1.5 *m*/*z*. Raw chromatograms were extracted without smoothing.

To ensure the accuracy of PCT concentration, the identification of each peptide was verified based on PRM LC-MS/MS data. The extracted ion chromatograms showed the coelution of two selected product ions, with the peptide of interest and its internal standard. The identification of the SAL peptide is presented in Figure 3.

Most product ions of the SAL peptide were identified in PRM data obtained from processed human serum. While the FHT peptide contains two residues of proline, which readily generates internal fragmentation from its N-terminal side, detected ions could not be attributed only to the primary peptide backbone fragmentation (see Figure S2, Supporting Information). Therefore, the two most intense product ions were selected, one for quantification and another for confirmation. For PRM data generated from triplicated analyses, the peak areas of selected transition were then extracted to establish a calibration curve based on isotope dilution and quantitative analysis.

#### Linearity

**Figure 4:**
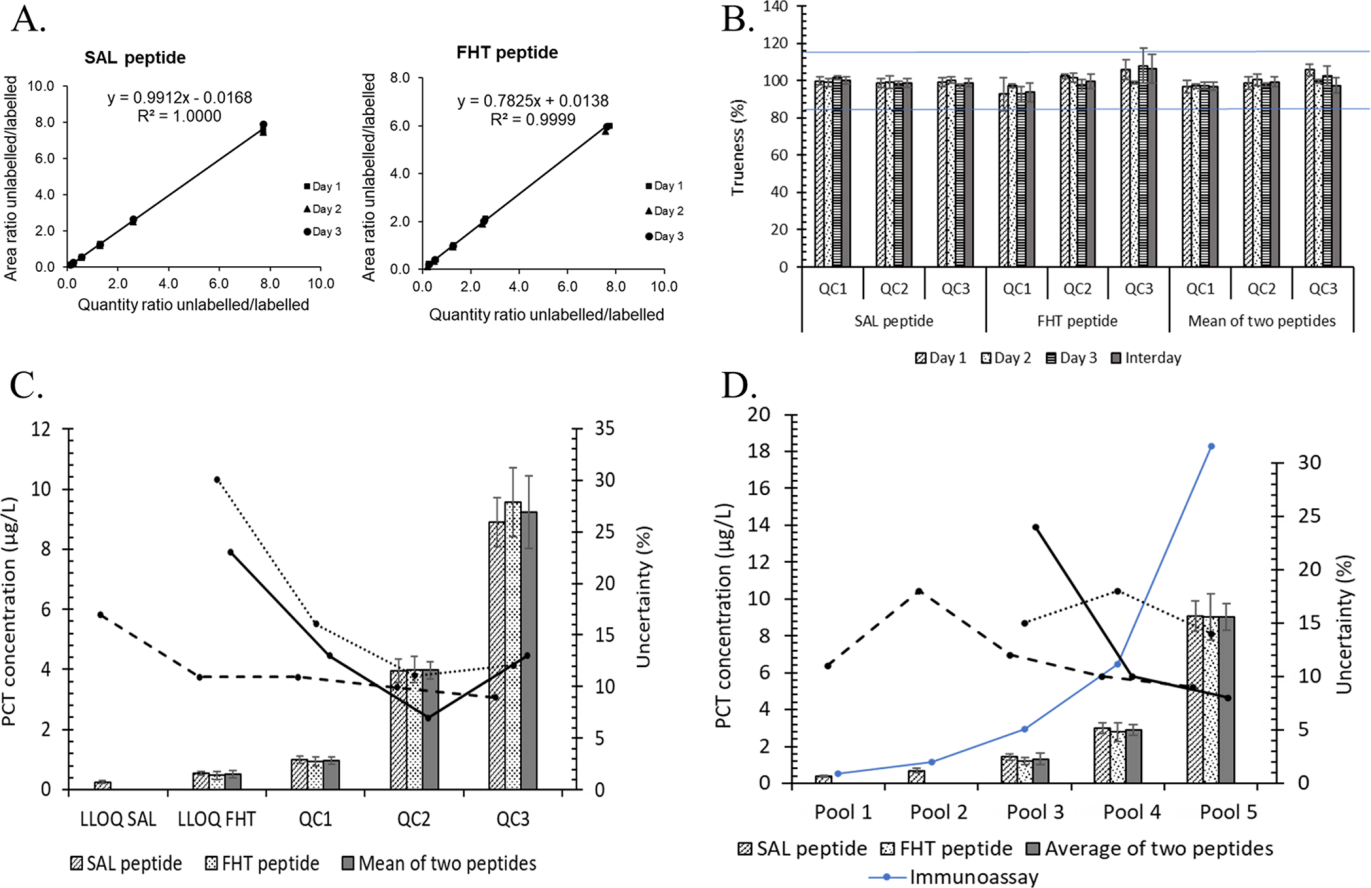
Method validation and estimation of uncertainties for PCT quantification in human serum. (A) Linearity of the signal response obtained with nonzero protein-based matrix-matched calibrators for the SAL peptide and FHT peptide. Linearity results obtained from three independent experiments (linearity equation) were obtained by averaging three independent experiments. (B) Intraday (*n* = 3) and interday (*n* = 3, 3 days) trueness and precision at three QC levels. Blue lines represent the acceptation limit ± 15% for the trueness value. Precision was expressed as an error bar. (C) Estimation of uncertainties of PCT concentration of QC materials. Expanded uncertainty was expressed by an error bar. Relative expanded uncertainty was presented by the dashed line (SAL peptide), dotted line (FHT peptide), and solid line (mean of two peptides). (D) Method application to quantify PCT concentration in patient pool samples compared to those obtained by immunoassay (in solid blue line). Expanded uncertainty was expressed by an error bar. Relative expanded uncertainty was presented by the black dashed line (SAL peptide), black dotted line (FHT peptide), and solid black line (mean of two peptides).

The regression model is linear over the range 0.25–13.74 μg/L for SAL and 0.47–13.74 μg/L for FHT (Figure 4A). The Pearson regression coefficient was above 0.998 for both peptides. Detailed data obtained for each peptide from three independent days are presented in Table S7 and Figure S3 of the Supporting Information.

#### Trueness and Precision

Trueness and precision of the method’s validation are presented in Figure 4B and detailed in Table S8. The intraday (*n* = 3) bias and interday (*n* = 3, 3 days) bias ranged from −2.8 to 1.6 and −1.2 to 0.2% for SAL and −7.3 to 8.0 and −6.2 to 6.3% for FHT. The intraday precision and intermediate precision (interday) were below 3.3 and 2.3% for SAL and 9.5 and 7.6% for FHT. For all QC materials, intraday bias was between −3.3 and +5.7%, and interday bias was between −3.0 and −0.7% for the mean concentration. Intraday precision was below 5.1%, and interday precision was below 4.0% for QC materials.

#### Lower Limit of Quantification

Extracted ion chromatograms from human serum at the LLOQ level are presented in Figure 3 and Figure S2. The LLOQ level was 0.25 μg/L for SAL and 0.47 μg/L for FHT. Therefore, PCT concentration was calculated by the average of two concentrations obtained from two peptides for concentration above 0.47 μg/L and by SAL only below this limit.

The mean bias and precision CV were 4.2 and 5.5%, respectively, for a concentration of 0.25 μg/L and −0.7 and 7.5% for a concentration of 0.51 μg/L.

#### Higher Limit of Quantification

The HLOQ quantification at a concentration above the highest calibrators was quantified after 20× dilution. It showed bias and precision of 1.6 and 2.3% for SAL and 5.5 and 0.2% for FHT. The method can quantify PCT for a concentration up to 132 μg/L.

#### Autosampler stability

Autosampler stability of 7 days at +7 °C was demonstrated for all QC levels (bias from the initial concentration <20%). The two peptide concentrations remained stable in the autosampler.

#### Carryover

No carryover was observed for the two peptides.

The present method uses a SIL protein as an internal standard that differs from the other LC-MS/MS methods developed to quantify PCT.^22−24^ The SIL protein added at the beginning of the sample preparation process is ideal for protein quantification with the bottom-up approach.^25,27^ It compensates for the bias caused by incomplete digestion or material loss during sample preparation and LC-MS/MS analysis.^24,26^ These limitations have been underlined in a previous study in which PCT was quantified through peptide-based calibration using SIL peptides as internal standards.^24^ A correction factor has been applied to compensate for digestion incompleteness and material loss before the digestion step. Moreover, the FHT peptide could not be used as a quantifier peptide as it may be subject to miscleavage not corrected by the approach used. In the present study, both endogenous and SIL-PCT are simultaneously proteolyzed. PCT quantification with low bias and high precision was archived without using a correction factor when quantifying both SAL and FHT peptides for concentrations above 0.47 μg/L, allowing to increase the specificity of the method. These two selected peptides are located in two different regions of PCT and are not in the same region of epitopes usually targeted by commercially available immunoassays.^24^

Furthermore, as reported in the literature, PCT is present under three different isoforms characterized by the cleavage of one or two N-terminal amino acids.^7^ Our method quantifies the total serum PCT, including these three isoforms as measured by most commercially available immunoassays.

The calibration range, HLOQ, and LLOQ of the method encompass the clinical range of PCT concentrations found in serum from sepsis or septic shock patients. Therefore, the candidate RMP is intended to be used to measure PCT in sepsis patients and support activities of the IFCC working group on the standardization of PCT assays (WG-PCT) to monitor the accuracy and comparability of immunoassay results and evaluate if the correlation between available immunoassays at different clinical cut-off concentrations is sufficient to conduct standardization. While the analytical sensitivity of the candidate RMP covers almost all of the ranges of concentrations measured by immunoassays, if the standardization of the PCT assay is confirmed to be needed and feasible, further studies are required to improve LLOQ to cover LLOQ of all commercial immunoassays (0.02–0.2 μg/L). This improvement could be achieved through instrumental developments (e.g., reducing LC flow rates and dimensions, using a more sensitive mass spectrometer) and improving the sample preparation step (e.g., using immunoenrichment). Miniaturization of sample handling could suffer from low reproducibility when analyzing low abundant analytes in complex and concentrated samples such as serum.^33^

### Application to the Measurement of Patient Samples

As a proof of concept to evaluate how results from the candidate RMP compare with those from immunoassays, the developed method quantification was further applied to five pools of patient samples on two independent experiments. The interassay precision ranged from 1.5 to 7.7% and from 6.5 to 10.5% for SAL and FHT, respectively (Figure 4D and Table S11, Supporting Information). The mean concentration was obtained with a precision below 5.1%. The concentration measured by immunoassay was higher than the one obtained by ID-LC-MS/MS, with a relative difference between ID-LC-MS/MS and immunoassay ranging from 18 to 55%.

This relative difference observed between LC-MS/MS and the immunoassay could be explained by differences in calibration and/or differences in specificity potentially caused by cross-reactivity issues. Although most PCT immunoassays employ two antibodies targeting different regions of PCT, it cannot be excluded that immunoassays measure other forms than the three full-length isoforms of PCT. However, it should be noted that only five samples were measured, and only one immunoassay was involved. Therefore, this did not allow making a definitive explanation and advocates for a larger study. Indeed, the result obtained from this assay could be different from the other assays because PCT assays were reported to employ different types of antibodies with different epitope specificities toward the multiple molecular forms of PCT.^7^ The correlation between commercial immunoassays and the candidate RMP should be established soon for all available immunoassays and not only Brahms PCT-sensitive Kryptor immunoassays. Also, a larger number of samples of proven commutability are required to establish a correlation, which was not demonstrated in the present study. These studies will be designed by IFCC WG-PCT and will help to confirm the magnitude and investigate the origin of differences observed in PCT concentration.

### Evaluation of Measurement Uncertainty

The uncertainty of the calibrator and the linear regression are presented in Tables S9 and S10 in the Supporting Information for each calibrator level. The relative expanded uncertainty of each concentration level of QC materials and pools of patient serum samples are presented in Figure 4C,D, respectively, and summarized in Table S12 of the Supporting Information. For all levels, the relative expanded uncertainty (*k* = 2) was below 18% and below 30% when using the SAL peptide and FHT peptide, respectively. The relative uncertainties were lower for the results obtained using the SAL peptide. The relative expanded uncertainty (*k* = 2) ranged from 7 to 18% for mean concentration, except for LLOQ FHT and Pool3 samples (about 24%). The relative contributions of the different components to the final uncertainty of individual concentration per peptide are presented in Figure S4 of the Supporting Information. The uncertainties associated with the value assignment of the primary calibrator (u_cal_), the linearity of the calibration curve (u_lin_), and the precision of measurements (u_prec_) appeared as the primary sources of measurement uncertainty. Their relative contributions varied depending on PCT concentration. The main contribution to the final uncertainty for low PCT concentrations was the uncertainty associated with the linear regression or the precision experiment, while for high PCT concentration, it was the uncertainty associated with the calibrator’s purity. The uncertainty of the precision experiment was higher for the FHT than for the SAL peptide.

To ensure that laboratory measurements are clinically usable, it has been recommended that no more than one-third of the maximum allowable uncertainty of routine assays should be consumed by higher-order references.^34^ In addition to the correct implementation of calibration traceability, the achievement of appropriate analytical performance specifications for RMPs and CRMs is essential but can be challenging for low abundant proteins like PCT. Relative expanded uncertainties of results obtained with our method are generally 7–18%, but they reached up to 24% in some cases (low PCT concentration level). These uncertainties are probably too high for assigning a target value to a standalone CRM but are acceptable if this remains an isolated event when the RMP is used to measure a panel of patient samples (e.g., correlation study between available immunoassays and candidate RMP). As high uncertainties might lead to a modest correlation between the candidate RPM and the immunoassays and might compromise the ability to properly evaluate the accuracy of immunoassays, reducing measurement uncertainties would be beneficial. The major source of uncertainty at low PCT concentration was the uncertainty associated with the linear regression (up to 54%): this source of uncertainty could be reduced by employing a narrow working concentration instead of a large concentration range (0.15–7.5 in mass ratio).^35^ This may be difficult to handle when a large number of samples of unknown PCT concentrations over an expanded range of concentration should be measured (correlation study between available immunoassays and candidate RMP) but very much manageable in the case of a value assignment of pairs of CRMs at a given concentration. It should also be noted that the high uncertainty observed in one pooled sample with low PCT concentration was caused by variability between concentrations of the two measured proteotypic peptides. As this was observed only in one pool of patient samples, a more extensive study involving a larger number of pooled samples and single donation samples, as the one planned to assess standardization feasibility, will help further demonstrate the magnitude and source of uncertainties at this PCT-level concentration.

## Conclusions

We developed and validated an ID-LC-MS/MS method for the SI-traceable quantification of PCT in human serum covering most clinical cut-off concentrations. We used a protein-based calibration strategy relying on a PCT recombinant protein as primary calibrator, and the corresponding isotope-labeled recombinant protein as an internal standard. Using recombinant protein as the primary calibrator and internal standard improved the method’s accuracy compared to a previously developed method based on peptide calibrators. A correction factor is not required anymore with the present method, as the protein-based internal standard accounts for incomplete digestion and material loss during sample preparation. The present method thus appears suitable to determine PCT concentration in external quality assessment materials and secondary CRMs that could be used to monitor the accuracy and comparability of commercially available immunoassays for PCT at clinically relevant concentrations. The candidate RMP will support the activities of IFCC WG-PCT and especially evaluate the feasibility for the standardizing PCT assays.

## Supporting Information Available

Description of the chemicals and reagents, preparation of solutions, method validation; tables: MS parameters for intact analysis and top-down protein analysis, amino acid sequences and PRM parameters of selected peptides, AAA results obtained from optimization and final optimized conditions, main impurities of the stock solution, detailed data for the calibration curve, intraday and interday bias and precision, uncertainties of the calibrators, uncertainties of the linear regression, peptide and PCT concentration of the patient serum samples, relative expanded uncertainty of measurement results; figures: Met-PCT [3–116] characterization by top-down analysis, identification of the FHT peptide for PCT quantification, linearity of PCT quantification by LC-MS/MS and uncertainty estimation (PDF)
