# Supplementary material for: Candidate High-Resolution Mass Spectrometry-Based Reference Method for the Quantification of Procalcitonin in Human Serum Using a Characterized Recombinant Protein as a Primary Calibrator
Source: Anal Chem. 2022 Mar 2;94(10):4146–54. doi: 10.1021/acs.analchem.1c03061 (PMC8928150; doi:10.1021/acs.analchem.1c03061)
Supplement: Supplementary file 1 — ac1c03061_si_001.pdf [file ac1c03061_si_001.pdf]

Supporting Information

A candidate high-resolution mass spectrometry-based reference method for the quantification of procalcitonin in human serum using a characterized recombinant protein as primary calibrator

Huu-Hien Huynh[a,b,#], Vincent Delatour[a], Maxence Derbez-Morin[a,c], Qinde Liu[d], Amandine Bœuf [a,†,*], Joëlle Vinh[b,†]

[a] Laboratoire National de Métrologie et d'Essais (LNE), Department of Biomedical and Organic Chemistry, 75724 Paris, France

[b] Biological Mass Spectrometry and Proteomics, SMBP, PDC UMR 8249 CNRS, ESPCI Paris, Université PSL, 75005 Paris, France

[c] Université Paris-Saclay, CEA, INRAE, Département Médicaments et Technologies pour la Santé (DMTS), SPI, 91191 Gif-sur-Yvette, France

[d] Chemical Metrology Division, Applied Sciences Group, Health Sciences Authority, 117528 Singapore, Singapore

[*] Email: amandine.boeuf@lne.fr; Phone: (33) 140 433 921

[†] *A.B. and J.V. contributed equally to this paper*

Table of Contents

List of documents:

Document 1: Chemicals and reagents…………………………………………………………………S3

Document 2: Preparation of solutions………………………………………………………...……… S4

Document 3: Method validation……………………………………………………………………..…S5

List of tables:

Table S1: Mass spectrometry parameters for intact protein analysis……………………………………..S7

Table S2: Mass spectrometry parameters for top-down protein analysis………………………………….S8

Table S3. Amino acid sequence, retention time, and PRM parameters for detecting the two selected peptides and internal standards. ……………………………………………………………………..S9

Table S4. AAA results obtained at different conditions of gas-phase hydrolysis, each condition with four processed replicates……………………………………………………………………………………S10

Table S5. AAA results obtained from 29 processed replicates over six independent gas-phase hydrolyses of the primary calibrator stock solution……………………………………………………………….S11

Table S6. Main impurities of the stock solution of primary calibrator obtained by HRMS………………S12

Table S7. Detailed data for calibration curves of each selected peptide. Results obtained by the mean of two transitions per peptide………………………………………………………………………………..S13

Table S8. Intra-day and inter-day bias and precision of 3 independent experiments on QC materials. Each experiment was performed on three processed replicates (n=3) at three different concentrations………S14

Table S9. Example of uncertainties of the calibrators spiked in serum (u_{cal})……………………….…..S15

Table S10. Example of uncertainties of the linear regression (u_{lin}) ………………………………….S16

Table S11: Individual peptide concentration and PCT concentration with inter-assay precision of the patient serum samples from two independent experiments…………………………………………...…S17

Table S12: Relative expanded uncertainty of measurement results (k=2) for 6 QC materials and 5 pools of human serum……………………………………………………………………………………………..S18

List of figures:

Figure S1. Met-PCT [3-116] characterization by top-down approach ……………………………..……S19

Figure S2. Identification of FHT peptide for PCT quantification in human serum……………………..S20

Figure S3: Linearity of PCT quantification by LC-MS/MS………………………………………..…...S21

Figure S4: Uncertainty estimation…………………………………………………………………S22

Document 1: Chemicals and reagents

The amino acid CRMs L-leucine (CRM 6012-a, 99.9±0.2 %), L-phenylalanine (CRM 6014-a, 99.9±0.2 %), L-proline (CRM 6016-a, 99.9±0.2 %) and L-valine (CRM 6015-a, 99.9±0.2%) were obtained from National Metrology Institute of Japan (NIMJ, Tsukuba, Japan). The labeled amino acids were purchased from Cambridge Isotope Laboratories, Inc. (Andover, MA, USA).

Sodium deoxycholate (SDC, cat# D6750-100G), ammonium bicarbonate (cat# 09830-500G), dithiothreitol (cat# 43819-1G), iodoacetamide (cat# I1149-5G), and Tween-20 (cat# P1379-100ML) were obtained from Sigma-Aldrich (St. Quentin Fallavier, France). Acetonitrile (ACN, cat# 0001204102BS), methanol absolute (cat# 0013684102BS), formic acid (cat# 00069141A8BS), and trifluoroacetic acid (cat# 00202341A8BS) (all LC-MS grade) were purchased from Biosolve Chimie (Dieuze, France). Acetic acid (cat# A113-50, LC-MS grade) was purchased from FisherChemical (Illkirch, France). Water was purified using a Milli-Q system (Millipore, MA, USA). Trypsin Gold (cat# V5280, MS grade) was obtained from Promega (Madison, USA).

Human serum (reference H4522) was purchased from Sigma Aldrich (St. Quentin Fallavier, France). The material was obtained from healthy male subjects and is noted blank serum afterward. Its PCT concentration was below the limit of detection (0.02 µg/L) of commercially available immunoassay (Roche Cobas).

Document 2: Preparation of solutions

All steps of preparation of solution were performed gravimetrically using calibrated precision balances. Therefore, its concentration is expressed by mass of compound of interest per mass of solution, except for final samples (calibrant, quality control) where its concentration is converted in µg/L in concordance with clinical use.

1. **Stock solutions and working solutions**

Individual primary stock solutions were gravimetrically prepared in human serum for Met-PCT [3-116] and SIL-protein Met-PCT at 12.18 µg/g and 14.23 µg/g, respectively. Solutions were aliquoted and stored at -80°C ± 10°C.

The two individual primary stock solutions of Met-PCT [3-116] and SIL-protein Met-PCT were diluted in human serum to obtain individual working solutions of unlabelled protein and labeled protein at 22.15 ng/g and 44.93 ng/g, respectively. Solutions were aliquoted and stored at -80°C ± 10°C.

2. **Protein calibrators and internal quality control materials**

Six points calibration curves were prepared by mixing an increasing volume of Met-PCT [3-116] working solution with a constant volume of SIL-protein Met-PCT working solution to obtain ratios ranging from 0.14 to 12.10 (corresponding to a PCT concentration from 0.25 µg/L to 13.74 µg/L).

Three quality control (QC) materials were prepared by mixing three different volumes of Met-PCT [3-116] working solution and a constant volume of SIL-protein Met-PCT working solution to reach an amount ratio of 0.51, 2.03, and 5.08 (corresponding to a PCT concentration of 0.88, 3.61 and 8.93 µg/L, respectively). The Met-PCT [3-116] working solutions used for calibration solutions and QC materials were prepared from two different aliquots of the individual primary stock solutions.

3. **Protein samples for dilution test**

PCT samples at 132 µg/L were prepared by diluting Met-PCT [3-116] working solution in human serum. This solution was then diluted with human serum by a factor of about 20 to prepare six PCT samples at 6.77 µg/L. The diluted PCT samples were then mixed with SIL-protein Met-PCT working solution to reach a concentration of 6.52 µg/L.

Document 3: Method validation

Linearity: The calibration curves were obtained with non-zero five to six calibrators depending on peptide by plotting the ratios of the peak areas of the peptides over their isotopically labelled counterparts against the corresponding ratios of the amounts of the peptides over their isotopically labelled counterparts. Linearity was evaluated by three sets of calibration samples prepared from three independent aliquots of Met-PCT [3-116] stock solutions that were then diluted in the serum samples obtained from healthy subjects. The linearity was evaluated according to the Pearson correlation coefficient, which was required to be >0.995. Besides, the accuracy of the quantification of each calibrant was evaluated by considering the calibrants as unknown samples. The bias between the measured concentration and the theoretical concentration determined gravimetrically should be within ± 20% for the LLOQ level and ± 15% for other levels according to the guideline of bioanalytical method validation from FDA and EMA [1,2]. For each batch, a maximum of one calibrator was excluded from the curve. The individual concentration per peptide was determined using its respective calibration curve; the mean concentration was calculated as the average of two individual concentrations.

Trueness and precision: The trueness and precision were evaluated in intermediate precision conditions of measurement using three processed replicates of QC materials at three concentration levels (about 1.0, 4.0 and 9.0 µg/L) over three independent experiments (intra-assay: N=3 per concentration level, inter-assay: N= 9 per concentration level). The concentration of each sample was determined using the calibration curve prepared on the same day. Trueness was evaluated by the bias between the measured concentration and the theoretical concentration determined gravimetrically. The precision was calculated as the coefficient of variation (CV) of the measured concentrations per peptide and the mean concentration of two peptides. Bias and precision (CV) should be within ±15 % according to the guideline of bioanalytical method validation from FDA and EMA [1,2].

Lower limit of quantification: The LLOQ was defined as the lowest concentration of the calibration curve with bias and precision within 20% according to the guideline of bioanalytical method validation from FDA and EMA [1,2]. The LLOQ was assessed by analyzing six processed replicate samples at 0.25 µg/L for SAL peptide and 0.5 µg/L for FHT peptide.

Higher limit of quantification: To determine whether a serum sample with a PCT concentration exceeding the highest concentration of the calibration curve can be diluted before being processed as described above, an experiment was conducted by diluting 20-folds with human serum obtained from healthy subjects. A spiked QC material (HLOQ) was gravimetrically prepared at a concentration of about 132 µg/L, which is 9-folds higher than the highest concentration of the calibration curve. The experiment was performed with six processed replicate samples. The bias and precision of the diluted samples should be within ± 15% according to the guideline of bioanalytical method validation from FDA and EMA [1,2].

Autosampler stability: The auto-sampler stability (7 C) of the final extract from QC materials was evaluated by comparing the concentration obtained at the beginning of the study (day 0) and those obtained 7 days later. The final extract was considered stable if the concentration was quantified within 20% of the initial concentration (day 0).

Carryover: Carryover is the ratio (in percentage) of peak area of the calibrator in blank sample acquisition, just after a prior injection of the calibrator at the highest concentration over the peak area of the LLOQ level. Carry-over should not exceed 20%.

1 EMA. ICH guideline M10 on bioanalytical method validation [Internet]. 2019 [cited 2020 Dec 17]. Available from: https://www.ema.europa.eu/en/documents/scientific-guideline/draft-ich-guideline-m10-bioanalytical-method-validation-step-2b_en.pdf.

2 FDA. Bioanalytical Method Validation Guidance for Industry [Internet]. 2018 [cited 2021 Jan 10]. Available from: https://www.fda.gov/files/drugs/published/Bioanalytical-Method-Validation-Guidance-for-Industry.pdf.

Table S1: Mass spectrometry parameters for intact protein analysis

Parameters	Value
Spray voltage (V)	3,500
Sheath gas flow rate*	35
Sweep gas flow rate*	1
Auxiliary gas heater temperature (°C)	200
Auxiliary gas flow rate	10
Capillary temperature (°C)	250
S-lens RF level*	50
Resolution	35,000
Scan range (m/z)	400-1,500
AGC target	$1^{E}6$

(*) arbitrary units

Table S2: Mass spectrometry parameters for top-down protein analysis

	Parameters	Value
	Spray voltage (V)	2,500
	Sheath gas flow rate*	10
	Sweep gas flow rate*	0
	Auxiliary gas heater temperature (°C)	140
	Auxiliary gas flow rate	5
	Capillary temperature (°C)	300
	S-lens RF level*	30
MS analysis	Orbitrap resolution	120,000
	Scan range (m/z)	500-2,000
HCD mode	Orbitrap resolution	120,000
	HCD collision energy (%)	35
	Isolation window (m/z)	1.8
	Filter at precursor level (m/z)	908-945
CID mode	Orbitrap resolution	120,000
	CID collision energy (%)	35
	Isolation window (m/z)	1.8
	Filter at precursor level (m/z)	908-945
EThCD mode	Orbitrap resolution	120,000
	ETD reaction time (ms)	2.5
	Isolation window (m/z)	1.8
	Filter at precursor level (m/z)	908-945
	SA Collision Energy (%)	35
UVPD mode	Orbitrap resolution	120,000
	UVPD activation time (ms)	100
	Isolation window (m/z)	1.8
	Filter at precursor level (m/z)	908-945

(*) arbitrary units

Table S3. Amino acid sequence, retention time and PRM parameters to detect the two peptides of interest and internal standards.

Proteotypic peptide	Retention time (min)	Precursor ions		Collision energy (eV)	Product ions		
		ion	m/z		ion	m/z	Type
SALESSPADPATLSEDEAR	19.2	$[M+2]^{2+}$	973.4529	35	y13+	1,371.6387	quantifier
					y10+	1,088.5218	qualifier
SALESSPADPATLSEDEAR[$^{13}C_6$,$^{15}N_4$]	19.2	$[M+2]^{2+}$	978.4570	35	y13+	1,381.6469	quantifier
					y10+	1,098.5301	qualifier
FHTFPQTAIGVGAPGK	14.9	$[M+3]^{3+}$	543.2912	20	y7+	585.3355	quantifier
					y3+	301.187	qualifier
FHTFPQTAIGVGAPGK[$^{13}C_6$,$^{15}N_2$]	14.9	$[M+3]^{3+}$	545.9626	20	y7+	593.3497	quantifier
					y3+	309.2012	qualifier

Table S4. AAA results obtained at different conditions of gas-phase hydrolysis.

Hydrolysis conditions	Concentration (μg/g) (CV %, n = 4) Leu	Phe	Pro	Val	Average of four amino acids (μg/g) (CV %)
110 °C, 40 h	814.4 (1.0)	808.7 (1.4)	804.7 (1.3)	814.4 (1.0)	819.5 (2.6)
130 °C, 24h	871.0 (1.6)	867.3 (0.5)	866.4 (2.1)	882.5 (3.3)	871.8 (0.9)
130 °C, 40h	878.2 (1.9)	864.4 (1.8)	881.2 (2.1)	941.3 (2.1)	891.3 (3.8)
130 °C, 72h	851.1 (3.5)	839.2 (3.0)	848.4 (4.9)	881.5 (5.4)	855.1 (2.1)
150 °C, 40h	775.6 (1.1)	773.5 (1.5)	783.2 (0.8)	873.6 (1.3)	805.5 (6.0)

Each condition being evaluated in four processed replicates using one of the aliquots provided by the manufacturer.

Table S5. AAA results obtained from 29 processed replicates over six independent gas-phase hydrolyses of the primary calibrator stock solution.

	Phe	Pro	Val	Leu
Met-PCT [3-116] mass fraction obtained by each AA (μg/g)	788	783	858	797
Standard deviation (μg/g)	78	73	74	70
Met-PCT [3-116] mass fraction by combining four AAs (μg/g)	807			
Expanded uncertainty U (coverage factor k=2) (μg/g)	72			
Relative expanded uncertainty U (coverage factor k=2) (%)	8.9%			

The primary calibrator stock solution was obtained by combining ten aliquots provided by the manufacturer in order to avoid inhomogeneity between aliquots.

Table S6. Main impurities of the stock solution of primary calibrator obtained by HRMS

Monisotopic mass (amu)	Retention time (min)	Peak area	Mass difference compared to Met-PCT (amu)	Area ratio
12749.11 (Met-PCT)	15.83	1.4E+09	-	100.00%
12,765.10	15.65	7.1E+07	15.97	5.07%
12,791.16	18.19	3.0E+07	42.05	2.14%
11,380.45	16.97	7.4E+06	-1,368.66	0.53%
12,778.08	15.61	7.3E+06	29.34	0.52%
12,732.05	15.78	6.4E+06	-17.06	0.46%
11,458.52	16.18	1.4E+06	-1,290.59	0.10%

Table S7. Detailed data of calibration curves for each selected peptide. Results obtained by averaging two transitions per peptide.

	Theorical concentration (µg/L)	Day 1	Day 2	Day 3	Mean	CV
SAL peptide						
Slope		0.9859	0.9641	1.0232	0.9911	3.0%
R2		0.9999	1.0000	0.9998	0.9999	
Bias (%)	0.23	1.2%	14.8%	14.2%		
	0.45	-2.8%	6.2%	14.3%		
	0.98	-0.9%	0.5%	-2.7%		
	2.27	-0.4%	-2.0%	-3.6%		
	4.55	0.8%	-0.7%	-0.2%		
	13.74	-0.1%	0.1%	0.1%		
FHT peptide						
Slope		0.7745	0.7666	0.7825	0.7746	1.0%
R2		0.9995	0.9994	0.9981	0.9990	
Bias (%)	0.45	4.4	-8.8	-2.8		
	0.98	-11.8	-0.2	-2.5		
	2.27	-1.4	0.0	-1.3		
	4.55	3.8	1.1	1.9		
	13.74	-0.3	-0.1	-0.2		

Table S8. Intra-day and inter-day bias and precision of 3 independent experiments on QC materials. Each experiment was performed on three processed replicates (n=3) at three different concentrations.

	Day 1			Day 2			Day 3			Inter-day		
	Concentration (µg/L)	Bias (%)	CV (%)	Concentration (µg/L)	Bias (%)	CV (%)	Concentration (µg/L)	Bias (%)	CV (%)	Concentrati on (µg/L)	Bias (%)	CV (%)
	SAL peptide											
QC1	1.00	-0.2	2.2	0.99	-0.9	2.2	1.02	1.6	1.0	1.00	0.2	2.0
QC2	3.95	-1.2	2.5	3.97	-0.8	3.3	3.93	-1.7	1.4	3.95	-1.2	2.3
QC3	8.93	-0.8	2.4	9.03	0.3	1.7	8.75	-2.8	1.2	8.90	-1.1	2.1
	FHT peptide											
QC1	0.94	-7.3	8.8	0.95	-2.8	0.9	0.93	-7.3	4.0	0.94	-6.2	5.0
QC2	3.95	2.4	1.3	4.09	1.5	2.5	3.90	-2.4	3.2	3.99	-0.3	3.8
QC3	10.09	6.0	5.2	8.90	-1.1	0.6	9.72	8.0	9.5	9.57	6.3	7.6
	Average of two peptides											
QC1	0.97	-3.3	3.3	0.97	-2.9	1.3	0.97	-2.9	1.9	0.97	-3.0	2.0
QC2	3.95	-1.2	3.3	4.03	0.6	3.1	3.89	-2.1	0.9	3.97	-0.7	2.7
QC3	9.51	5.7	3.0	8.96	-0.4	1.0	9.23	2.6	5.1	9.24	-2.6	4.0

Table S9. Example of uncertainties of the calibrators spiked in serum (u_{cal})

Calibrator	Concentration (µg/L)	Uncertainty (µg/L)	Relative uncertainty (k=1) (%)
Cal 6	13.75	0.61	4.45
Cal 5	4.53	0.20	4.45
Cal 4	2.25	0.10	4.45
Cal 3	0.90	0.04	4.45
Cal 2	0.45	0.02	4.46
Cal 1	0.26	0.01	4.48
Cal 0	0	-	-

Table S10. Example of uncertainties of the linear regression (u_{lin})

Calibrator	Quantity ratio unlabeled/labeled peptide	Peak area ratio unlabeled/labelled peptide	Uncertainty	Relative uncertainty (k=1) (%)
Cal 6	7.74	7.62	0.0195	0.3
Cal 5	2.61	2.59	0.0082	0.3
Cal 4	1.28	1.26	0.0079	0.6
Cal 3	0.56	0.54	0.0086	1.6
Cal 2	0.24	0.23	0.0091	3.9
Cal 1	0.12	0.12	0.0093	7.7
Cal 0	0	0		--

The uncertainty of the linear regression was obtained using Excel (Microsoft Office). The uncertainty associated with the measurement result obtained from the linear calibration was calculated by the random error associated with both the intercept and the slope.

Table S11: Individual peptide concentration and PCT concentration with inter-assay precision of the patient serum samples from two independent experiments

	IA Conc. (µg/L)	ID-LC-MS/MS						Relative difference between ID-LC-MS/MS and IA(%) *
		SAL peptide		FHT peptide		Average of two peptides		
		Conc. (µg/L)	CV (%)	Conc. (µg/L)	CV (%)	Conc. (µg/L)	CV (%)	
Pool1	0.52	0.43	1.5	< LLOQ	-	-	-	-18.0%
Pool2	1.16	0.71	7.7	< LL0Q	-	-	-	-38.7%
Pool3	2.93	1.46	5.9	1.24	6.5	1.35	3.1	-53.9%
Pool4	6.48	3.01	2.9	2.82	10.5	2.92	5.1	-55.0%
Pool5	18.30	9.10	2.5	9.05	7.7	9.07	4.3	-50.4%

(*) relative difference = (LC-MS/MS-Immunoassay)/Immunoassay

IA= ImmunoAssay, Conc.= concentration

Table S12: Relative expanded uncertainty of measurement results (k=2) for 6 QC materials and 5 human serum pools.

		SAL peptide		FHT peptide		Average of 2 peptides	
Level	N	Conc (µg/L)	$U_{k=2}$ (%)	Conc (µg/L)	$U_{k=2}$ (%)	Conc (µg/L)	$U_{k=2}$ (%)
LLOQ SAL	5*	0.25	17	< LLOQ	-	-	-
LLOQ FHT	6	0.54	11	0.47	30	0.51	23
QC1	9	1.00	11	0.94	16	0.97	13
QC2	9	3.95	10	3.99	11	3.97	7
QC3	8*	8.90	9	9.57	12	9.24	13
HLOQ	6	136.80	10	127.20	10	132.00	12
Pool1	3	0.43	11	< LLOQ	-	-	-
Pool2	3	0.71	18	< LLOQ	-	-	-
Pool3	6	1.46	12	1.24	15	1.35	24
Pool4	6	3.01	10	2.82	18	2.92	10
Pool5	6	9.10	9	9.05	14	9.07	8

(*) one sample was excluded due to sample loss during sample handling. N= number of measurements; Conc=concentration

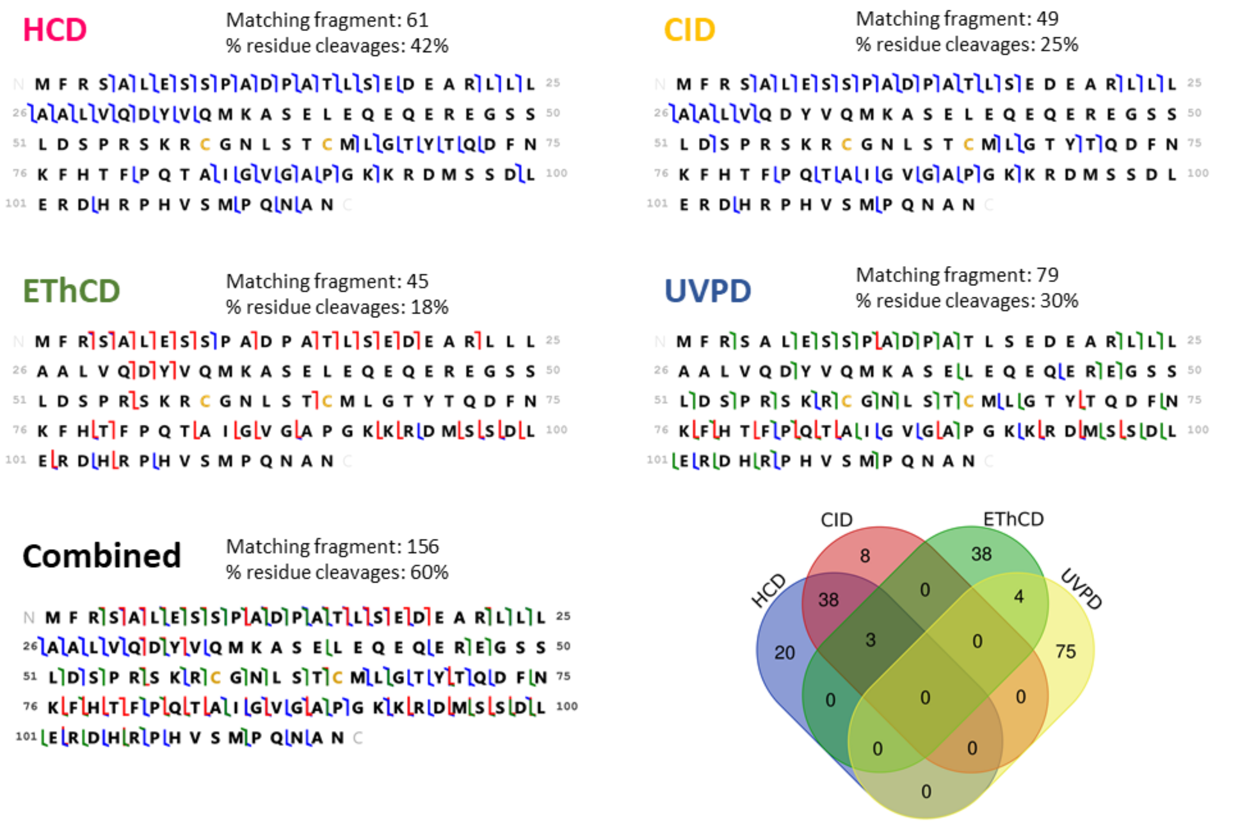

Figure S1. Met-PCT [3-116] characterization by Top-down approach

MS/MS was performed on the Orbitrap Eclipse by targeting the most intense charge state of MPCT (m/z 912.2293, z=14) in nanoESI infusion mode. The a and x fragments ions are indicated in green, b and y fragment ions in blue, and c and z fragment ions in red. When combining the results from the four fragmentation modes, the sequence coverage is 60%. Deconvoluted data were analyzed with ProsightLite software. Venne diagram was generated from https://www.bioinformatics.psb.ugent.be

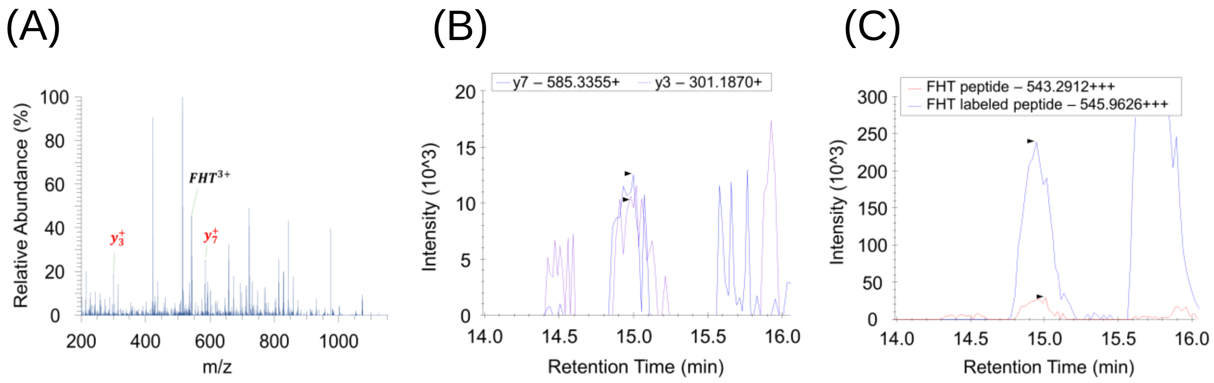

Figure S2. Identification of FHT peptide for PCT quantification in human serum. (A).MS/MS PRM spectrum of targeted precursor ion FHT^{3+} (selected product ions y_7^+ and y_3^+ for quantification in red) in processed human serum spiked with a PCT at 5 µg/L; (B) Extracted ion chromatograms obtained when measuring blank serum spiked with a PCT at 0.25 µg/L showing a coelution of two selected product ions; (C). Extracted ion chromatograms obtained when measuring blank serum spiked with a PCT at about 0.25 µg/L and labeled PCT at 1.5 µg/L showing a coelution of FHT peptide and its internal standard obtained. Precursor ions were isolated within an isolation window of 1.5 m/z. Raw chromatograms were extracted without any smoothing processing.

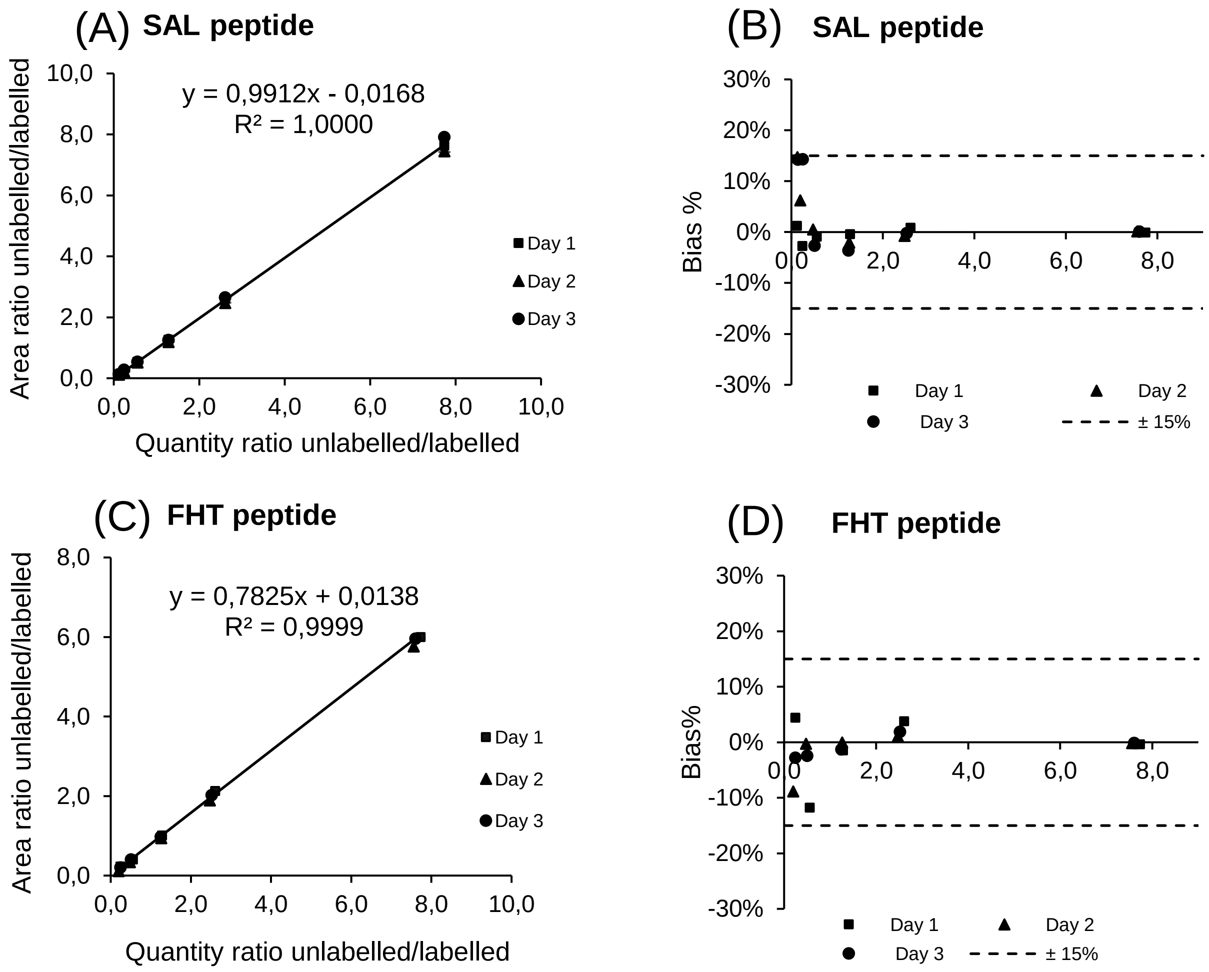

Figure S3: Linearity of PCT quantification by LC-MS/MS

(A) Linearity of the signal response for SAL peptide obtained with non-zero six points serum-based calibrators from three independent experiences (linearity equation was obtained by the average of results obtained from three independent days); (B) Percentage of deviation (%) of the back-calculated ratio compared to the theoretical quantity ratio of the SAL peptide; (C) Linearity of the signal response for FHT peptide obtained with non-zero five points serum-based calibrators from three independent experiences (linearity equation was obtained by averaging results obtained from three independent days); (D) Percentage of deviation (%) of the back-calculated ratio compared to the theoretical quantity ratio of the FHT peptide. Deviation % from all calibrator levels were between ±15%.

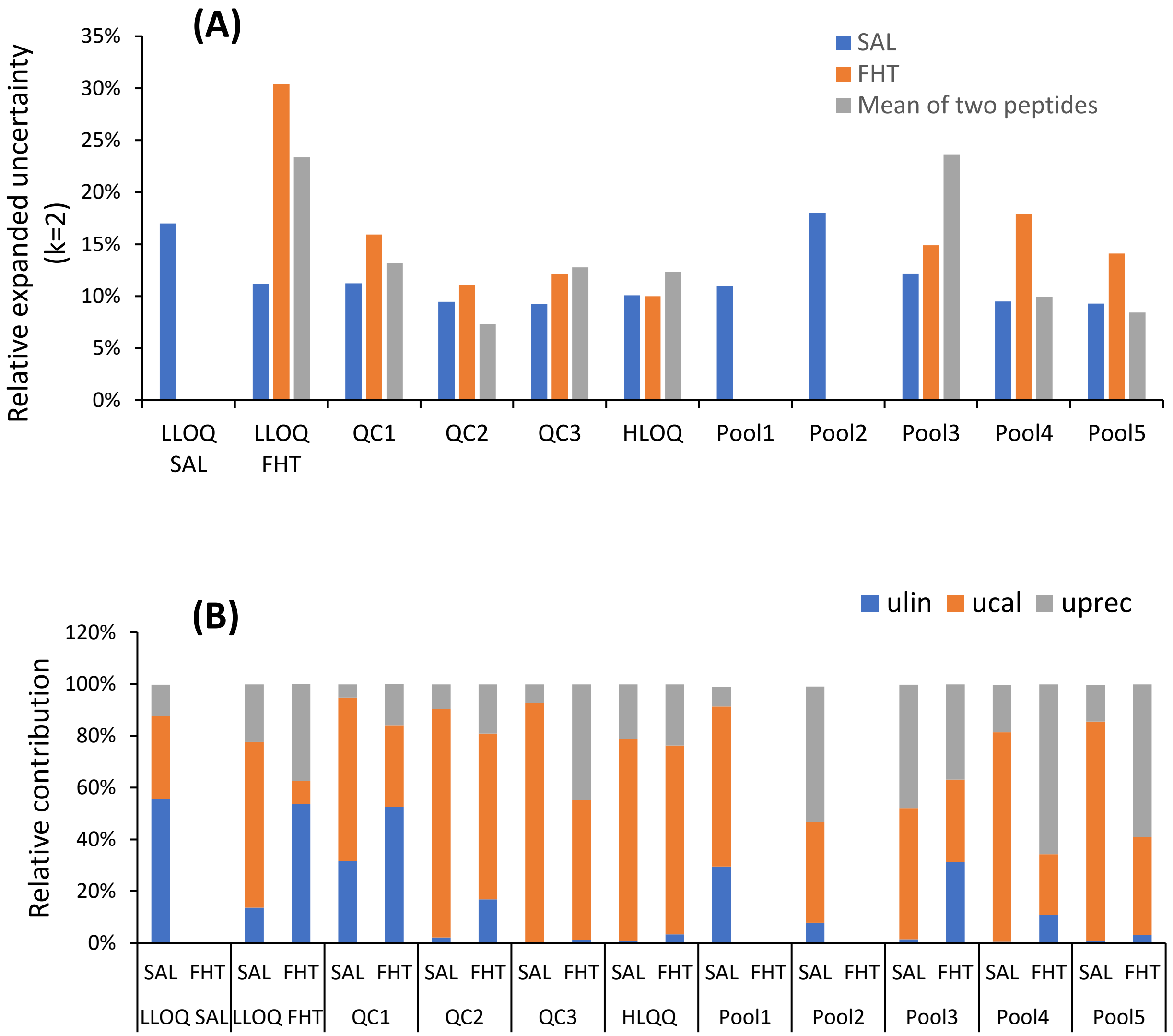

Figure S4: Uncertainty estimation. (A) Relative expanded uncertainties of the individual peptide results and the final results. (B) Relative contribution of the different components to the final uncertainty of individual peptide results: uncertainty of the calibrator (u_{cal}), the uncertainty of the linear regression (u_{lin}) and the precision experiment (u_{prec}), the uncertainty of gravimetric preparation of samples (u_{sam}) is negligible.
